# Reproxalap ‐ A Novel Treatment Option for Allergic Conjunctivitis – A Comprehensive Narrative Review

**DOI:** 10.1002/hsr2.72492

**Published:** 2026-05-31

**Authors:** Sanila Mughal, Roshanay Ejaz Awan, Hur Abbas, Zahra Sania, Ashna Habib, Syeda Kaneez Sakina, Amina Mahmud, Mohammed Mahmmoud Fadelallah Eljack

**Affiliations:** ^1^ Department of Medicine Dow University of Health Sciences Karachi Pakistan; ^2^ Department of Medicine, Women Medical and Dental College Khyber Medical University Pakistan Abbottabad Pakistan; ^3^ Faculty of Medicine and Health Sciences, Kassala Teaching Hospital, Kassala University of Bakht Alruda Ad Duwaym Kassala State Sudan

## Abstract

**Background and Aims:**

Allergic conjunctivitis is a prevalent, immune‐mediated hypersensitivity disorder of the ocular surface affecting 10%–20% of the global population. Current therapeutic options, while effective to some extent, are often limited by side effects and inconsistent efficacy. Despite its widespread impact, allergic conjunctivitis receives comparatively less clinical attention than other chronic allergic conditions. This review aims to assess the therapeutic potential and clinical efficacy of Reproxalap, a novel ophthalmic solution targeting reactive aldehyde species (RASP), for the treatment of allergic conjunctivitis.

**Methods:**

A narrative review was conducted analyzing the pharmacological profile of Reproxalap and its clinical performance in phase 2 and phase 3 clinical trials. These trials evaluated the drug's safety, tolerability, and efficacy based on symptom relief, particularly ocular itching and inflammation.

**Results:**

Reproxalap demonstrated early‐onset and broad‐spectrum anti‐inflammatory activity through RASP inhibition. By trapping free aldehydes and modulating inflammatory mediators like cytokines and chemokines, it significantly reduced ocular inflammation. Clinical trial data indicated statistically significant improvement in itching scores and sustained symptom relief in patients with allergic conjunctivitis. The drug also showed a favorable safety profile, with minimal adverse events reported.

**Conclusion:**

Reproxalap represents a first‐in‐class RASP inhibitor with promising efficacy and safety for the treatment of allergic conjunctivitis. Its unique mechanism of action and encouraging clinical trial outcomes support its potential as a valuable therapeutic alternative that may improve patient outcomes and could also be applicable in other inflammatory ocular conditions.

## 1 Introduction

Allergic conjunctivitis (AC) is one of the most common forms of allergic eye disease characterized by immune‐driven hypersensitivity of the entire ocular surface, secondary to environmental allergens [1]. It has become increasingly prevalent in recent years, affecting approximately 10%–20% of the people globally [2], and up to 40% of the people in the United States [3], with nearly one‐third of affected people being left undiagnosed and undocumented [4]. Various factors, including heredity, inflammatory response, air pollution, atopy, exposure to pollens, and pets contribute to the development of AC [5]. It manifests initially as itching, tearing, conjunctival and eyelid swelling, and redness that gradually progresses over hours to photophobia, ocular pain, visual impairment, and discharge [2]. It affects almost 30% of children [6] and can occur concomitantly with asthma, allergic rhinitis, eczema, and other atopic conditions. Although a benign condition, if left untreated, it could lead to more severe forms, including keratoconjunctivitis, scarring of the eye, ulcerations, bacterial or viral keratitis, pannus, and progression to infective conjunctivitis, thereby increasing the risk of serious secondary infections [5].

Allergic conjunctivitis is classified into five subtypes: seasonal allergic conjunctivitis (SAC), perennial allergic conjunctivitis (PAC), vernal keratoconjunctivitis (VKC), atopic keratoconjunctivitis (AKC), and giant papillary conjunctivitis (GPC), with SAC and PAC comprising 95% of cases. Although SAC and PAC contribute to a higher incidence, these are milder forms in contrast to relatively rarer forms, AKC and VKC that contribute to the most severe forms of the disease [2]. Nevertheless, the management of AC gets less attention than other chronic and allergic diseases and therefore, is associated with substantial morbidity and mortality. According to the current guidelines provided by ICOR, the management of allergic conjunctivitis starts with self‐treatment and non‐prescription medications, progressing to pharmacological treatments if needed. The current treatment modalities include antihistamines, topical decongestants, corticosteroids, immunomodulators, allergen immunotherapy, and surgical resection [7].

Reactive aldehyde species (RASP) are pro‐inflammatory molecules that act in periods of inflammation to increase the pro‐inflammatory signaling cascade through sustained release of cytokines and inflammasomes. Recently, inhibiting the actions of RASP has become a potential therapeutic target for AC. RASP inhibitors are novel class of small molecular compounds that bind to free aldehyde targets of RASP, inhibit the pro‐inflammatory signaling cascade, and also decrease the levels of histamine, which results in attenuation of signs and symptoms of inflammation. Reproxalap is a recently developed, first‐in‐class RASP inhibitor for the treatment of AC and has demonstrated remarkable efficacy and safety in phase 2 and 3 trials [8]. In this review, we aim to provide a comprehensive overview of the clinical efficacy and safety of Reproxalap in the treatment of AC and other ocular diseases as demonstrated by the clinical trials and its implications in the clinical world.

## 2 Methodology

This narrative review discusses the relevant literature, including the clinical trials on Reproxalap for the treatment of AC and other ocular diseases. In June 2024, literature search was conducted from the time of inception to May 10, 2024, using the databases PubMed, Clinical Trials, and Google Scholar. The search terms included “Reproxalap,” “Allergic Conjunctivitis,” “ADX‐102,” and other related keywords, including treatment, safety, and efficacy. We included articles in the English language. Abstract‐only articles, commentaries, letter to the editor, and articles in languages other than English were excluded from this review.

## 3 Pathophysiology of Allergic Conjunctivitis

The conjunctiva provides a mucosal covering around the eyelids and ocular surface. Owing to its large surface area and accessibility, it is a common site for direct antigen deposition and triggering the allergic cascade. Allergic conjunctivitis is induced by an allergen‐induced inflammatory response, primarily a type 1 hypersensitivity reaction mediated by immunoglobulin E (IgE). When an allergen reaches the conjunctiva of a sensitized individual, it interacts with IgE attached to sensitized mast cells, setting off a chain reaction that produces the clinical allergic manifestation. Th2‐cells produce cytokines that induce IgE production by B‐cells, which bind to the mast cell membranes and allergens, provoking the secretion of inflammatory mediators [9]. Reactive aldehydes contribute to ocular inflammation by inducing oxidative stress, modifying cellular proteins, and triggering inflammatory cascades, leading to symptoms such as redness, itching, and conjunctival swelling [9, 10, 11].

After exposure, the immediate or early phase of the allergic cascade starts seconds to minutes later and lasts for 20–30 min in a clinical setting [10]. During this phase, mast cell activation results in increased histamine, tryptase, prostaglandins, and leukotrienes in the tears, causing symptoms like redness, weeping, pruritus, chemosis, and a papillary reaction. Mast cell degranulation also activates vascular endothelial cells, causing them to express chemokines and adhesion molecules such as vascular cell adhesion molecule (VCAM) and intercellular adhesion molecule (ICAM). Other chemokines released include eotaxin, interleukin (IL)−8, monocyte chemoattractant protein (MCP), regulated upon activation of normal T cell expressed and secreted (RANTES) chemokines, and macrophage inflammatory protein (MIP)−1 alpha [9, 11].

The late phase starts with the infiltration of the inflammatory cells into the conjunctiva, followed by the expression and activation of various proteins and chemoreceptors of activated leukocytes, leading to the chronic phase of the allergic reaction. Chemokines play a crucial role in the framework of late‐phase allergic reactions like tryptase, chymase, IL‐4, and other cytokines expressed by conjunctival mast cells. IL‐4 also promotes IgE production from B cells, adhesion molecules, and chemokine secretion, and monitors Th2 lymphocyte development. Leukotrienes, cytokines, and chemokines are released by activated eosinophils [12, 13]. Furthermore, they release the neurotoxin, eosinophil peroxidase, eosinophil cationic protein, and eosinophil major basic protein [14, 15].

Numerous of these products are epitheliotoxic, which means they might lead to ocular problems such as infiltrates and ulcers [16, 17]. The expressions of corneal keratocytes, conjunctival fibroblasts, and epithelial cells facilitate the recruitment of inflammatory cells and the start of the late‐phase reaction [12, 13].

## 4 Current Treatment Options for Allergic Conjunctivitis

Despite the availability of highly effective treatment options for acute forms of ocular allergies, the treatment for the chronic forms of this condition is still debatable [2]. The current treatment options available for AC are as follows:

1. Non‐pharmacologic therapy
  The signs and symptoms of AC have been treated using a wide range of systemic and topical medications [2, 3, 4, 5, 10, 18, 19]. However, non‐pharmacologic therapy should always be used initially, and topical treatments should be used in tandem [2, 4, 10]. Like all other allergic diseases, the best defense against AC, is allergen avoidance which is especially important in PAC and SAC and in AKC and VKC when there is a history of allergen exposure [2, 3, 4, 5, 10, 18, 19, 20]. Other non‐pharmacological approaches, such as the use of sunglasses, cold compresses, isotonic saline drops, and cold and preservative‐free artificial tears or ointments, also provide relief from various symptoms of ocular allergies [2, 3, 4, 5, 10, 18, 19, 20]. However, these approaches are frequently insufficient to produce long‐term, and occasionally even short‐term, symptom alleviation [3].
2. Drug therapy
  Among drug therapies for AC, topical antihistamines, mast cell stabilizers, and dual‐action drugs stand as the first‐line treatment [2, 10]. Other drugs approved for the treatment of AC have been listed in Table 1. Although various drug classes are approved for AC, each drug presents distinct limitations and drawbacks, hence necessitating the development of novel drugs for the treatment of this disease. Common antihistamines effectively reduce conjunctival inflammation [2, 3, 5, 10, 18], however they have minimal effect on other proinflammatory mediators generated during an allergic reaction. Moreover, the short duration of action of these antihistamines necessitates repeated dosages which decreases the patient compliance [3]. Oral antihistamines like loratadine, desloratadine, and fexofenadine are effective [2] but can cause systemic side effects like drowsiness, confusion, and urinary retention [2, 3, 10], along with dry eyes, which may worsen the conjunctivitis symptoms [2, 3, 4, 20]. Unlike first‐generation antihistamines, second‐generation antihistamines exhibit longer duration of action, with improved safety profile, and their availability in topical solutions makes it a favorable therapeutic option for treating AC [3, 4, 10]. The use of oral antihistamines is complemented with topical ocular therapies such as artificial tears to attenuate symptomatic relief in AC. However, administration of oral antihistamines has been associated with cardiotoxicity in some cases [3, 4, 18, 20].
  Mast cell stabilizers do not provide immediate symptomatic relief [3] but work best when used proactively to prevent future allergen‐induced reactions [4]. Applying these drugs topically as a loading dose before allergen exposure is most effective [2, 3, 4, 5, 18]. However, mast cell stabilizers have the common side effects of irritation of the ocular surfaces and headaches [2]. In addition, lodoxamide is not approved by the FDA for long‐term use [5]. Dual‐acting agents like olopatadine and ketotifen combine immediate H1 receptor blockage with the prophylactic role of mast cell stabilizers thus requiring less frequent dosing and improving compliance [2, 3, 4, 10, 18]. However, these drugs can cause burning, stinging, blurring of vision, and an unacceptable aftertaste [4, 21].
3. Topical decongestants
  Topical decongestants are non‐prescription medications that cause vasoconstriction in the conjunctiva, reducing hyperemia and edema. Prolonged use can result in a rebound in hyperemia and decreased effectiveness over time due to tachyphylaxis. Moreover, minimal side effects such as epiphora, lacrimal puncta obstruction, mydriasis, stinging, and systemic hypertension have also been reported with chronic use [2, 3, 4, 10, 20, 21]. When these treatments fail, NSAIDs are occasionally administered as adjunctive medications. This family of drugs can alleviate itching, redness, tearing, and foreign body sensation [2, 3, 10]. While beneficial in the short term, extended use of NSAIDs can cause serious side effects like corneal keratitis, ulceration, or perforation [3, 10].
4. Steroids
  Steroids work to treat AC by lowering mast cell proliferation, inflammatory cytokine production, and cell‐mediated immune responses [3, 10]. Owing to the higher risk of increased intraocular pressure (IOP) and cataract development, these drugs are typically prescribed for short‐term use [2, 3, 4, 10, 20]. Owing to the immunosuppressant effect of steroids, they can also lead to higher infection rates and poor wound healing if used for prolonged periods of time [2, 4, 18].
5. Other treatment modalities
  Other treatment modalities for AC include allergen‐specific immunotherapy, immunomodulators, and biological response modifiers [2, 3, 4, 10, 19]. Immunotherapy is the only therapy that can deliver long‐term benefits by downregulating the Th2 response and overexpressing regulatory T cells, diminishing reactions to allergen exposure [2, 10]. It can be used in children, though those younger than 6 years, may face challenges due to the rigorous desensitization regimen [2]. Among other side effects of this treatment listed in Table 1, the long‐term schedule of immunotherapy can lead to poor patient compliance [10]. Immunophilins, a class of topical calcineurin inhibitors, have recently gained clinical approval for chronic allergic conditions like GPC, AKC, and VKC [4, 19, 20]. However, their use is associated with an increased risk of herpes simplex keratitis and rare incidents of skin cancer and lymphoma have also been reported [20]. Omalizumab, a humanized monoclonal antibody, has shown effectiveness in treating VKC and AKC but is not yet authorized for ocular allergies [22]. Another biological agent dupilumab, an inhibitor of the IL‐4 and IL‐13 pathways, has not been examined in AC, but conjunctivitis has been reported as an adverse effect of this medication [10].
6. Surgery

**Table 1:** Current treatment options of allergic conjunctivitis (AC).

Drug class	Drugs	Mechanism of action	Dosing	Effects	Side effects	Indications	Age indications	Contraindications
Topical anti‐histamines [2]	Emedastinedifumarate (emadine) (0.05%) Levocabastine (0.05%) Pheniramine maleate	H1‐receptor Antagonists	One drop, QID	Decrease permeability of venules, Reduced chemotaxis of inflammatory cells, reduced itching, Increased sensitivity	Stinging in the eyes, Keratitis	First line treatment for all forms of AC	> 3 years	N/A
Oral antihistamines (second generation) [10, 18]	Loratidine Desloratidine Fexofenadine Cetirizine	H1‐receptor Antagonists	Loratidine – 10 mg orally OD for adults Desloratidine – 5 mg orally OD Fexofenadine – 60 mg orally BD or 180 mg orally OD Cetirizine – Oral administration: 5–10 mg once daily for allergic rhinitis and urticaria Intravenous administration: 10 mg once daily for acute urticaria	Same as topical antihistamines with systemic effects	Sedation and urinary retention with first‐generation drugs, reduction in tear production causing dry eyes, Potential cardiotoxicity	Allergic Rhino‐ Conjunctivitis, only in SAC/PAC, VKC, and AKC when other systemic symptoms present; not in GPC and CBC	Not recommended for children less than or equal to 4 years of age.	Relative contraindications include pregnant and lactating women, hypertension, cardiovascular diseases, urinary retention, renal or hepatic dysfunction, and increased IOP. Caution in QT prolongation or concomitant use with other QT prolonging drugs (59)
Mast cell stabilizers [3]	Nedocromil sodium (2%) Sodium cromoglycate (4%) Lodoxamidetromethamine (Alomide) (0.1%) Pemirolast potassium (0.1%)	Prevents calcium mobilization which inhibits mast cell degranulation	All one drop QID except Nedocromil which is given one drop BID	Prevents the release of pre‐formed inflammatory mediators from mast cells; decreased redness, hyperemia, itching, and redness.	Burning and irritation in the eyes, Headaches. Lodoxamide is not FDA‐approved for long‐term use	Prophylaxis of AC, Mast cell stabilizers in nasal sprays or aerosols for the treatment of concomitant allergic rhinitis and asthma	Lodoxaminde: > or equal to 4 years Cromoglycate: > or equal to 5 years Pemirolast: > or equal to 3 years	Hypersensitivity to the drug or any of the components of the formulation (60)
Dual action agents [10]	Olopatadine (0.1%) (Patanol) Olopatadine (0.2%) (Pataday) Olopatadine (0.7%) (pazeo) Ketotifen fumarate (0.025%) (Zaditor or Alaway) Bepotastinebesilate 1.5% (Bepreve) Alcaftadine 0.25% (Lastacaft) Epinastine 0.05% (Elestat) Azelastine 0.05% (Optivar)	Both H1‐receptor antagonist and mast cell stabilizing effect	BID Daily Daily BID to TID (one drop every 8–12 h) BID Daily BID BID (1 drop in the affected eye twice per day)	Combined immediate effects of antihistamines and long‐term effects of mast cell stabilizers, Inhibition of eosinophil migration, Relive itching associated with allergic conjunctivitis	These drugs use benzalkonium chloride as a preservative which induces toxic effects on ocular surfaces, Bitter taste (azelastine), headache, stinging, and burning	First line for all forms of AC, Superior to both anti‐histamines or mast cell stabilizers when given alone	> or equal 3 years > or equal to 16 years > or equal to 2 years > 3 years > or equal to 3 years > or equal to 3 years > or equal to 3 years > 3 years	Contraindicated in the case of hypersensitivity reactions. Soft contact lens wearers should remove their lens before using these drops, and should not out them back till after atleast 15 min (because benzalkonium chloride can discolor soft contact lens) (61,62)
Topical ocular vasoconstrictors [21]	Naphazoline hydrochloride Tetrahydrozoline hydrochloride	Alpha‐adrenergic agonists (typically alpha 1)	Maximum QID, short term Maximum QID, Short term	Alpha‐adrenergic activation causes vasoconstriction which reduces redness and edema	Follicular reaction, Conjunctivitis, contact dermatitis, mydriasis, Tachyphylaxis, Potential for misuse by patients, systemic hypertension	In VKC and AKC in combination with anti‐histamines	Children > 14 years of age	Narrow‐angle glaucoma, Contra‐indicated with MAO inhibitors, In children under 14 years of age
Topical NSAIDS [4]	Diclofenac 0.1% (VoltarenOphtha) Ketorolac 0.4% (Acular LS) and 0.5% (Acular) Nepafenac 0.1% (Nevanac) Bromfenac 0.7% (Prolensa)	COX inhibitors which ultimately reduce prostaglandin release	All are given as one drop QID	Reduce itching To minimize mucus secretion, cellular infiltration, erythema, and chemosis.	Stinging, Ocular hypertension, Keratitis, corneal ulceration, or perforation	For the relief of ocular itching and pain associated with seasonal AC	All > or equal to 18 years of age	Asthma, Nasal polyps
Topical cortico‐steroids [3]	Fluorometholone acetate 0.1% (FML) Prednisolone acetate 1.0% (Pred Forte) Loteprednoletabonate 0.2% (Lotemax, Alrex) Loteprednoletabonate 0.5% (Lotemax)	Phospholipase A2 inhibition, Decreased T cell activation, reduction in histamine release	BID BID QID QID	Anti‐inflammatory actions, reduced activity of inflammatory cells, and lowering of most ocular signs and symptoms	Immunosuppression leads to increased infection rate and delayed healing, Cataracts, ocular hypertension, and glaucoma	Moderate to severe forms of ocular allergy, acute exacerbations, and keratopathy	> 2 years All ages > or equal to 18 years > or equal to 18 years	Contraindicated in patients with viral infections, general steroid contraindications
Oral leukotriene inhibitors [20]	Montelukast sodium	Leukotriene receptor antagonist	There are no precise recommendations about the dosing and age at which montelukast should be used for allergic conjunctivitis. However, because allergic conjunctivitis and allergic rhinitis frequently overlap, the same age cutoff is indirectly applied to both conditions. The suggested dosages for patients with seasonal allergic rhinitis are 10 mg for those over 15, 5 mg for those between the ages of 6 and 14, and 4 mg for those between the ages of 2 and 5. (63)	Reduce nitric oxide levels in the conjunctiva	Most commonly headaches, neuropsychiatric disorders, cough, dyspepsia, and abdominal discomfort. In children, it causes otitis, sinusitis, pharyngitis, and laryngitis	SAC and PAC; in AKC, along with Aspirin	Allergic rhinoconjunctivitis is the term for allergic conjunctivitis and AR that occur simultaneously. Conjunctivitis and AR typically coexist (64) The FDA has approved montelukast for the treatment of seasonal AR in adults and children 2 years of age and up. Additionally, it is authorized for the treatment of perennial AR in adults and children 6 months of age and up. (64,65). Category B in pregnancy (64)	Contraindicated in patients with hypersensitivity to the drug or any component of the drug, contraindicated in severe asthma, and care should be taken in PKU patients
Immuno‐modulators [20]	Cyclosporine A (0.05%) emulsion Tacrolimus ointment (0.03%) Pimecrolimus cream (1%)	Calcineurin inhibitors, called immunophillins, Blocks IL‐2 production, inhibiting IL‐2‐mediated proliferation of T cells.	One drop BID BID	Steroid‐sparing effect	Increased susceptibility to herpes simplex keratitis, rarely cause skin cancer, or lymphoma (tacrolimus and pimecrolimus)	Chronic forms of VKC, AKC and GPC not responding to steroids; cyclosporine in treatment of dry eyes; tacrolimus in treatment of lid eczema for VKC, oral forms in treatment of refractory disease	Tacrolimus drops 0.03% in children 2–15 years of age Patients ages 16 or older: tacrolimus drops 0.03% or 0.1% Patients 2 years or older: pimecrolimus cream 1% or topical tacrolimus ointment	Drug allergy to this class of drugs.
Immuno‐therapy [4]	Sublingual immunotherapy (SLIT) Subcutaneous immunotherapy (SCIT)	The known allergen is progressively introduced in higher amounts to establish desensitization which results in downregulation of immune response mediated by Th2 cells and activation of T‐regs.	Daily allergen injections in SLITor SLIT may be taken daily either as quick tablets or drops that are kept under the tongue for at least 1 min before being swallowed. Once or twice weekly allergen injections in SCIT then reduced frequency to once monthly allergen injections Both are given in two phases: an induction phase of 5–8 months, and a maintenance phase of 3–5 years.	Produce immune tolerance; nasal symptoms are relieved more than ocular symptoms	Systemic allergic reaction rate is higher in SCIT as compared to SLIT Oral swelling and itching and abdominal pain with SLIT, Risk of anaphylaxis	Chronic allergic rhino‐conjunctivitis, in SAC/PAC, VKC. AKC when hypersensitivity against a known allergen; when failure of first‐line treatments, and when the patient experiences moderate‐to‐severe symptoms that interfere with normal daily activities or sleep despite regular and adequate medication and/or avoidance tactics	Difficult to treat children < 6 years of age because of long‐term, strict plan	During pregnancy severe, uncontrolled asthma Active systemic autoimmune disorder or malignant neoplasia Relative contra‐indications topical or systemic beta‐blocker therapy, cardiovascular disorders, severe psychiatric diseases, immunodeficiency diseases, and previous history of systemic reaction to immunotherapy
Biologicals [2]	Insunakinra (EBI‐005) Liftitegrast (Shire Pharmaceuticals) Omalizumab Dupilumab	First two are IL‐1 receptor antagonists; liftitegrast is also lymphocyte function antigen‐1 (LFA‐1) antagonist Omalizumab is an anti‐IgE monoclonal antibody that targets the FCεR3 component of unbound IgE. (10) Dupilumab is a human monoclonal antibody, which acts as IL‐13 and IL‐4 inhibitor (10)	Insunakinra—for dry eye disease, it is used in the concentration of 5 or 20 mg/mL, 1 drop TDS for 6 weeks (66), for allergic conjunctivitis: 5 mg/mL topical TDS (67) Lifitegrast: Ophthalmic solution containing lifitegrast 50 mg/mL (5%). One drop BD (68) Omalizumab is available as • A single‐dose prefilled syringe containing an injectable solution of omalizumab (75 mg/0.5 mL).• A single‐dose prefilled syringe containing an injectable solution of omalizumab (150 mg/mL) • A single‐dose vial containing 150 mg of lyophilized omalizumab powder for reconstitution. The patient's body weight and serum total IgE levels are typically used to establish the dosage and frequency of omalizumab. In the US, a total serum IgE level between 30 and 700 IU/mL is advised. The recommended range for total serum IgE levels in Europe is between 30 and 1500 IU/mL for adults and children 12 years of age or older, and less than 1300 IU/mL for children 6–12. (69) No specific dosing regimen available for the use of dupilumab for allergic conjunctivitis. But it is generally given subcutaneously, and is available in the form of a pre‐filled syringe and a pre‐filled pen. Further dosing depends on the disease being treated (70) The initial suggested dose of dupilumab is two SC injections at separate injection sites, followed by one injection at varied places every 2–4 weeks, depending on the patient's age and weight. (71)	Decrease ocular symptoms such as pruritis, redness, and discomfort	Cough, upper respiratory tract infections, and nonfasting blood glucose elevation were the most frequent nonocular AEs associated with insunakinra. There were few documented eye adverse events. (66) Dysgeusia and local ocular discomfort are the most frequent side effects of lifitegrast. Other symptoms include headache, sinusitis, hypersensitivity reactions, blurred vision, and swollen tongue. (72) Omalizumab‐anaphyaxis, Injection site responses, viral infections, upper respiratory tract infections, sinusitis, headaches, and pharyngitis are among the side effects of omalizumab that have been reported in clinical trials. Others: transient hairloss and churgg strauss disease, parasitic infectionsIt can cross the placental barrier and cause low birth weight in infants exposed to the drug via maternal usage during pregnancy. (69) • Dupilumab‐associated ocular surface disease as a side effect of using dupilumab for the treatment of AC (2,20) Joint aches, allergic reactions such asthma and shortness of breath, and nasopharyngitis, headaches, epitaxis, herpes viral infections, psoriasis and psoriatic arthritis, helminth infections, and immunogenicity are among the rare but potentially dangerous side effects of dupilumab. (70,71)	Insunakinra and lifitegrast—for the relief of ocular surface symptoms of AC (2) Omalizumab‐off label use for resistant VKC (10,57,58) Dupilumab‐being studied for management of AKC (59)	Insunakinra‐only studied on adult patient > or equal to 18 years of age so far (72) and that link Lifitegrast‐safety below age 17 not yet established (68) Omalizumab‐minimum age of use depends on the disease being treated. But mostly > or equal to 6 years of age (73) Dupilumab‐ Only adults and pediatric patients who are at least 2 years old should use the pre‐filled pen. The pre‐filled syringe is indicated for use in adults and pediatric patients ≥ 6 months of age. For pediatric patients ≥ 12 years of age, administration by or under the supervision of an adult is advised. It is not advised for pediatric patients between the ages of 6 months and 12 to self‐administer. (70) For individuals 6 months of age and older with moderate‐to‐severe atopic dermatitis, the FDA has approved dupilumab. Additionally, it is authorized as an additional maintenance treatment for moderate‐to‐severe asthma in children 6 years of age and older who have an eosinophilic phenotype or are dependent on oral corticosteroids. Dupilumab is also authorized for individuals with eosinophilic esophagitis who are 12 years of age or older and weigh at least 40 kg. Nevertheless, dupilumab is not FDA‐approved for use in children with prurigo nodularis or chronic rhinosinusitis accompanied by nasal polyposis. (71) Age indications in context of allergic conjunctivitis have not been found	Lifitegrast and Omalizumab and dupilumab are contraindicated if previously a severe hypersensitivity reaction to the drug is noted in the patient (68,69,70) The FDA's current guidance solely advise against administering live virus vaccinations to dupilumab patients (71).

In addition to medical treatment, eye surgery can be performed in resistant cases of VKC and AKC [2, 5]. Severe VKC with corneal ulcers can be treated with papillae resection, often combined with autologous conjunctiva, amniotic membrane, or mucous membrane grafting [2, 23]. Resection is needed for sub‐epithelial deposits in VKC. Surgery for AKC may address scarring on the eyelids and conjunctiva. Complications like sub‐capsular cataracts and severe ocular surface diseases require advanced procedures such as superficial keratectomy, limbal transplantation, or keratoprosthesis installation [2].

**Figure 1:**
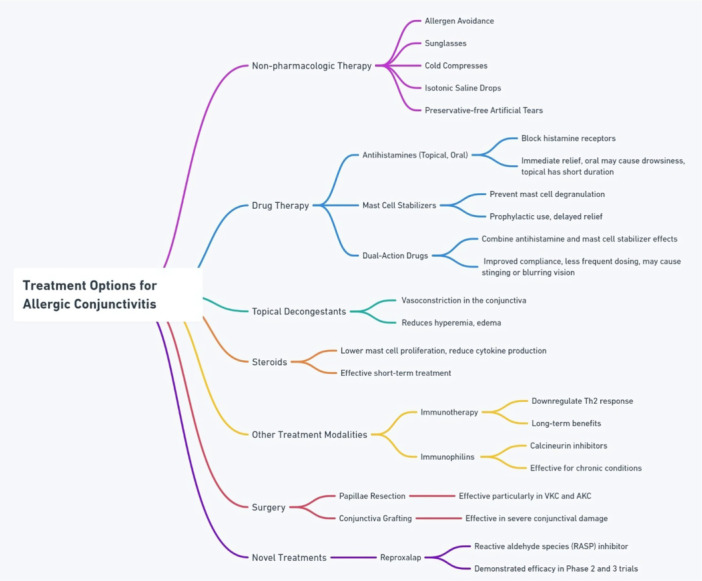
Summary of the current treatment options for allergic conjunctivitis and their key characteristics.

Despite significant advances in managing AC, limitations persist, such as the standardization of medication doses and the development of long‐term agents with improved patient compliance. While essential medications exist for AC, there is no adequate treatment for persistent and severe forms like AKC and VKC [21]. The safety and proper dosing of corticosteroids, the most effective topical anti‐inflammatory medicines, remain serious concerns. There are no guidelines or consensus among scientific bodies on the indications and treatment duration with topical formulations of immunomodulators such as cyclosporine and tacrolimus [21]. Owing to the complex, multifactorial nature of the disease, individuals with AC still report persistent of ocular symptoms with the use of combination therapies. Moreover, the use of more potent agents such as immunosuppressants and topical corticosteroids in resistant cases increases the risk of serious adverse events, thereby limiting their long‐term use [3]. Hence, these issues highlight the importance of new pharmacological agents for the management of AC. Figure 1 summarizes the current treatment modalities for AC with their key characteristics.

## 5 Reproxalap as a Novel Alternative

The development of Reproxalap ophthalmic solution offers a promising treatment for AC, dry eye disease (DED), noninfectious anterior uveitis (NAU), and Sjögren–Larsson syndrome. Formerly known as ADX 102 or NS‐2, Reproxalap (C12H13ClN2O, MW 236.697) is a first‐in‐class RASP inhibitor developed by Aldeyra Therapeutics, providing early‐onset and broad anti‐inflammatory effects [24]. Unlike conventional agents that primarily target specific inflammatory pathways, Reproxalap demonstrated the ability to trap free aldehydes, reduce inflammation, accelerate healing, protect cellular components from aldehyde damage, and lower the risk of scarring or fibrosis [25]. Furthermore, it exhibits remarkable tolerability with no serious adverse effects reported in clinical trials as compared to the currently available treatments. Its higher patient compliance rates highlight its potential to effectively address healthcare needs. Thus, Reproxalap stands out among all therapeutic agents as a novel alternative for AC, demonstrating its potential to improve patient care without severely impacting the patient's quality of life.

## 6 Mechanism of Action

**Figure 2:**
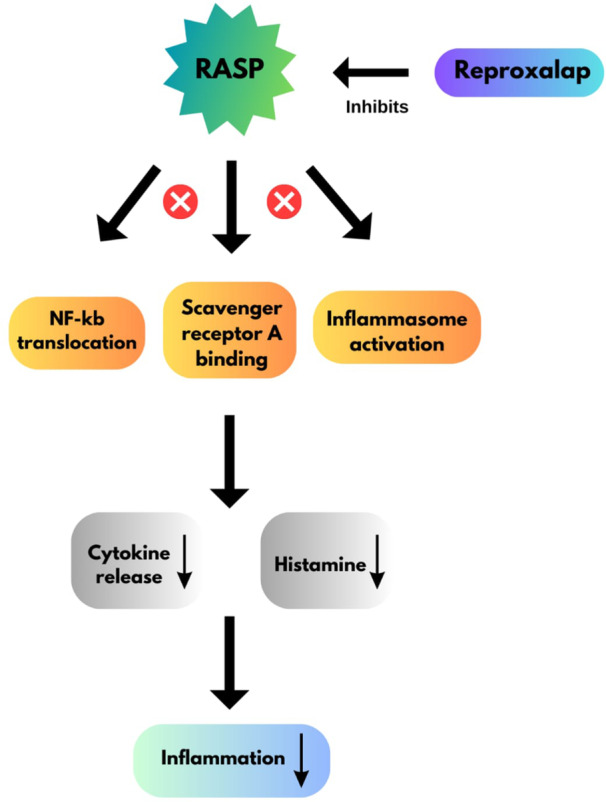
Mechanism of action of reproxalap.

Reproxalap, an innovative topically applied RASP inhibitor, has shown clinical efficacy in NAU [26], DED [24], and AC [27]. RASP molecules act as gatekeepers of inflammation, regulating it at the top of the cascade. Glycation, amino acid oxidation, and lipid peroxidation are some of the in vivo sources of RASP; however, non‐enzymatic free radical processes are the main source [28]. RASP causes modifications to ion channels and enzymes and generates pro‐inflammatory substrates such as cyclooxygenase‐2 and tumor necrosis factor‐alpha, producing new electrophilic aldehydes, which in turn cause amplification of the cascade, cellular necrosis and apoptosis, and damage to DNA [29]. RASP generates DNA adducts as a Michael addition of deoxyguanosine by 4‐hydroxynonenal (4‐HNE) [30, 31]; Microsomes, mitochondria, and lipid‐rich membranes often contain the largest RASP accumulations. Increased risk factors for cardiovascular disease and diabetes [32, 33], Behçet's disease [34], allergies [35], uveitis [36], and DED [37] are linked to elevated levels of RASP. RASP inhibitors prevent cytokine production by inhibiting NF‐kB translocation, scavenger receptor A binding, and inflammasome activation [3]. Histamine and pro‐inflammatory cytokine levels are decreased by this inflammation‐blocking [3]. The anti‐inflammatory properties of Reproxalap are being studied for use in treating ocular disorders such as AC, DED, and NAU [38]. Prior to the release of cytokines, RASP is upregulated during inflammation, which leads to the potentiation of nuclear factor kappa B, inflammasomes, and other proteins [39, 40]. RASP modulates the structure and function of proteins in an analog manner [41] based on the amount of binding. The post‐histaminic pathophysiology of allergies, including cellular infiltration and other sub‐acute physiological features of AC, is facilitated by RASP‐mediated activation of inflammation [42]. Reproxalap is a first‐in‐class treatment modality that targets RASP and modifies protein systems implicated in the inflammatory cascade indirectly, rather than directly activating or inhibiting a particular protein [8]. The mechanism of action of Reproxalap has been illustrated in Figure 2.

## 7 Clinical Trials

### 7.1 Phase 2 Trials

Reproxalap has demonstrated favorable activity and efficacy in phase 2 trials [27, 43, 44] that was confirmed in three phase 3 trials. The main results of clinical trials are comprehensively summarized in Table 2.

**Table 2:** Clinical trials evaluating the efficacy of reproxalap.

Phase of clinical trial	Author, year	Type of clinical trial	Total number of participants	Dosage	Trial assessment	Trial duration	Key efficacy endpoints	Safety *n* (%)
Phase 2 trial [44]	Cavanagh et al., 2022	Randomized, double‐masked, vehicle‐controlled	196	0.25%, 0.5%	To assess the activity of 0.25% and 0.5% reproxalap with the vehicle in an environmental controlled chamber	28 days	Improvement in ocular tearing (pooled reproxalap groups *p*‐value < 0.001; *p*‐value = 0.005 for reproxalap 0.25%; *p*‐value < 0.0001) Improvement in ocular itching (pooled reproxalap groups *p*‐value = 0.026; *p*‐value = 0.058 for each reproxalap 0.25% and 0.5%) Improvement in ocular redness and swelling was not statistically significant.	Reproxalap 0.25%: 13 (25%) Reproxalap 0.5%: 16 (31%)
Phase 2 trial [43]	Clark et al., 2021	Randomized, double‐masked, vehicle‐controlled	70	0.25%, 0.5%	To assess patient‐reported ocular itching and tearing scores	EEC allergen exposure	Improvement in ocular tearing (*p*‐value < 0.0001 for each reproxalap 0.25% and 0.5%) Improvement in ocular tearing (*p*‐value < 0.0001 for each reproxalap 0.25% and 0.5%) Improvement in conjunctival redness (*p*‐value < 0.0001 for each reproxalap 0.25% and 0.5%)	Reproxalap 0.25%: 47 (70%) Reproxalap 0.5%: 54 (78%)
Phase 2b trial [27]	Gomes et al., 2018	Randomized, double‐masked, vehicle‐controlled, parallel group	154	0.1%, 0.5%	To assess patient‐reported ocular itching after administration of 0.1% and 0.5% topical ocular ADX‐102 in the conjunctival allergen challenge	14 days	Improvement in ocular itching at 30 min post‐challenge (*p*‐value = 0.0347 for 0.5% ADX‐102) Improvement in ocular itch area under the curve (*p*‐value = 0.02 for ADX‐102 0.1%, *p*‐value = 0.004 for ADX‐102 0.5%)	Not reported
Phase 3 INVIGORATE trial [8]	Starr et al., 2023	Quadruple‐masked, vehicle‐controlled, crossover, sequence‐randomized	95	0.25%	To assess patient‐reported ocular itching and redness in a well‐controlled allergen chamber over a period of 3.5 h	EEC allergen exposure	Improvement in ocular itching (*p*‐value < 0.001) Improvement in conjunctival redness (*p*‐value < 0.001) Improvement in ocular tearing (*p*‐value < 0.001)	66 (69%)
Phase 3 INVIGORATE 2 trial [45]	Salapatek et al., 2024	Replicate, randomized, double‐masked, crossover design, vehicle‐controlled	131	0.25%	To assess patient‐reported ocular itching and conjunctival redness	EEC allergen exposure	Reproxalap was superior to vehicle for patient‐reported ocular itching, redness, tearing (*p*‐value < 0.05, *r* > 0.9, 95% CI)	Not reported
Phase 3 ALLEVIATE trial [46]	Clark et al., 2021	Parallel‐group, double‐masked, randomized phase 3 trial	318	0.25%, 0.5%	To assess the activity of 0.25% and 0.5% reproxalap with the vehicle in an environmental controlled chamber	EEC allergen exposure	Improvement in ocular itching at 60 min post‐challenge (mean 0.7, SD 0.8, *p*‐value = 0.003 for reproxalap 0.25%; mean 0.5, SD 0.7, *p*‐value 0.03 for reproxalap 0.5%) ≥ 2‐point responders from peak baseline ocular itching score for reproxalap 0.25% (*p*‐value = 0.0005; odds ratio = 2.36) and for reproxalap 0.5% (*p*‐value = 0.02; odds ratio = 1.81) Baseline‐adjusted time to ocular itching score 0 was faster for both reproxalap 0.25% (*p*‐value < 0.0001; hazard ratio = 2.14) and for reproxalap 0.5% (*p*‐value = 0.001; hazard ratio = 1.91)	Reproxalap 0.25%: 31 (28.4%) Reproxalap 0.5%: 41 (41.0%)

In a multicentre, phase 2b clinical trial [27], 154 patients with AC received different doses of ADX‐102 topical ophthalmic solution or vehicle drops. Those who received 0.5% ADX‐102 showed statistically lower ocular itching scores compared to the vehicle group at 10‐, 20‐, and 30‐min post‐exposure to allergens. The odds of responders experiencing a 1‐point improvement in itching were over 3 times higher in the ADX‐102 groups compared to the vehicle group. These results demonstrate ADX‐102's efficacy in providing immediate and sustained relief from ocular itching with a favorable safety profile.

In a single‐center phase 2 trial [43], Reproxalap (0.25% and 0.5%) demonstrated its clinical utility in 70 patients with SAC. Patients experienced a decrease in patient‐reported ocular itching, tearing, and conjunctival redness scores with prophylactic administration of Reproxalap. Notably, the 0.25% Reproxalap group showed a slower worsening of symptoms and a significantly lower proportion of patients with > 2‐point increase in symptoms score compared to the control group (hazard ratios [95% confidence interval] of 0.67 [1.0, 2.2] and 0.51 [1.0, 3.7] for itching and redness, respectively). This is one of the first data from a controlled trial that demonstrates Reproxalap's effectiveness in preventing symptoms before allergen exposure and treating AC during or after exposure. Another vehicle‐controlled, phase 2 trial assessed the efficacy of Reproxalap in 52 patients with SAC using the same study design [44]. The outcomes were consistent with the single‐center phase 2 trial [43].

### 7.2 Phase 3 Trials

The ALLEVIATE trial [46], is a multi‐center phase 3 clinical trial that evaluated the efficacy of Reproxalap in two concentration groups (0.25% and 0.5%). The trial comprised of a total 318 patients with seasonal post‐acute AC. Results showed significant decreases in mean ocular itching scores in both Reproxalap groups. The 0.25% concentration showed the most notable improvement, with many patients reporting no itching from 10 to 60 min post‐challenge. Additionally, faster symptom resolution to an ocular itching score of 0 was observed with the 0.25% concentration.

The INVIGORATE trial assessed Reproxalap 0.25% against a vehicle in 95 patients with AC. Results mirrored the phase 2 trial [43], showing that Reproxalap reduced ocular symptoms during allergen exposure, with lower itching scores across all time points over 3.5 h, indicating rapid and sustained effects. These results were further confirmed in the INVIGORATE 2 trial [45], conducted in an environmental exposure chamber, further proving Reproxalap 0.25%'s efficacy in reducing and relieving ocular symptoms in different subjects.

Therefore, the consistency across findings of all phase 2 and 3 trials provides strong evidence of Reproxalap, especially of 0.25% dosed ophthalmic solution, in alleviating ocular signs and symptoms of AC and indicates that these findings are reproducible reliable, and generalizable to the wider population.

## 8 Other Therapeutic Uses of Reproxalap

### 8.1 Non‐Infectious Anterior Uveitis

Several non‐clinical studies have demonstrated that Reproxalap and related molecules reduce ocular inflammation, and neutrophil infiltration and thereby decrease the oxidative stress and corneal inflammation [47, 48]. Similarly, a phase 2 randomized control trial (RCT) [26] also concluded that patients with NAU who were treated with Reproxalap either in combination or as monotherapy recovered faster to grade 0 flare as compared with patients treated with conventional prednisolone acetate ophthalmic suspension. Similarly, the efficacy of Reproxalap for NAU has also been confirmed in another study [49]. Additionally, Reproxalap showed a better safety profile. The conventional treatment with prednisolone is associated with elevation of ≥ 10 mmHg intraocular pressure, whereas it was not associated with treatment with Reproxalap. Thus, Reproxalap offers a promising alternative to conventional corticosteroid‐based treatments for NAU.

### 8.2 Dry Eye Disease

Treatment with Reproxalap significantly improved ocular discomfort, including dryness, stinging, burning, and grittiness scores on the ocular discomfort 4‐symptom questionnaire (ODS4Q). Schirmer's Test, tear osmolarity, and Lissamine Green staining scores also demonstrated significant improvement [24, 50, 51, 52, 53]. Patients experienced symptom relief as early as 1–2 weeks after starting therapy, with benefits increasing over time, indicating a modest dose response [24, 50, 53]. Therefore, Reproxalap is a promising alternative for moderate‐severe DED with a remarkable safety profile. Most clinical trials report no serious adverse events leading to drug discontinuation, though mild, self‐limiting irritation at the instillation site was noted. Nevertheless, a phase 2b trial reported two serious adverse effects (vertigo and angina pectoris), although deemed unrelated to the drug, it indicates the need for further long‐term studies in diverse populations to confirm Reproxalap's safety.

## 9 Side Effects

In clinical trials, Reproxalap has shown outstanding tolerance and safety profiles. No significant changes were observed in visual acuity, fundus examinations, vital signs, or lab results [8, 26, 43, 44, 46]. The most commonly reported treatment‐emergent adverse event (TEAE) in the 89‐patient phase 3 INVIGORATE trial was mild and transient installation site irritation, which affected 66 patients (69%) receiving reproxalap compared to four patients (4%), who were in the vehicle group. Transient mild chemosis was seen in 8.8% of patients. Rarely, two or fewer patients reported other TEAEs like lacrimation, eyelid swelling, pruritus, discomfort, hyperemia, and asthenopia [20]. One participant reported decreased visual acuity, considered possibly drug‐related but not clinically significant [46]. In the Phase II trial by Mandell et al., no major adverse events were reported; all adverse events were mild to moderate and resolved independently [26]. TEAEs were noted in 47 patients (70%) after 0.25% Reproxalap exposure, 54 patients (78%) after 0.5% Reproxalap exposure, and six patients (9%) after exposure to the vehicle in Clark et al. phase II trial and no patient had a major adverse event [43]. The Cavanagh et al. study revealed TEAEs in 25% and 31% of patients exposed to 0.25% and 0.5% Reproxalap, respectively, with no major adverse events. Overall, the most common side effect was temporary discomfort during instillation. Reproxalap has a very good tolerance profile, as shown by the numerous clinical trials assessing its safety profile. Compared to corticosteroids, which pose a risk of elevated intraocular pressure and cataracts, Reproxalap demonstrated a superior safety profile with no major long‐term adverse effects reported in clinical trials. These findings underscore Reproxalap's potential as a well‐tolerated treatment for ocular diseases, supporting further research and potential therapeutic use.

## 10 Discussion

The efficacy and safety of Reproxalap have been evaluated for the treatment of conditions, including AC, DED, NAU, and Sjögren–Larsson syndrome. Two different Reproxalap concentrations, 0.25% and 0.5%, have been implicated in the management of AC. In a phase 2 trial [27], topical ocular ADX‐102 was used in two control groups, 0.1% and 0.5% concentration where the latter showed a greater improvement in the ocular itching scores as compared to the vehicle group. In another phase 2 trial [43], both the concentrations of Reproxalap (0.25% and 0.5%) were evaluated, demonstrating a decrease in ocular itching, tearing, and conjunctival redness scores with prophylactic administration. As post hoc testing revealed no statistical difference between the two Reproxalap groups, the lowest effective dose selected was 0.25% [43]. Similarly, in another phase 2 trial [44], the graphs representing eyelid swelling, ocular tearing, and itching except for ocular redness, tend to favor the 0.5% group the most. Nevertheless, there was no statistically significant difference in the outcomes across both Reproxalap groups compared to the vehicle group [44]. In the phase 3 INVIGORATE trial, with only 0.25% concentration group, ocular itching, and redness scores remained in favor of this concentration group [8]. Similarly, the phase 3 ALLEVIATE Trial evaluated both concentrations and identified a notable reduction in mean ocular itching scores in both the groups, with 0.25% group exhibiting earlier symptom alleviation [46]. Moreover, when used for DED, 0.1% and 0.25% concentrations of Reproxalap have been assessed in trials where the outcomes remained in favor of 0.25% concentration [24]. Hence, Reproxalap (0.25% concentration) indicates an effective future treatment for AC.

To better contextualize the role of Reproxalap in clinical practice, we directly compared it to both non‐pharmacological and pharmacological treatment options for AC, highlighting its unique advantages, as well as remaining limitations. Reproxalap shows better efficacy, and safety profile with greater patient compliance and mild adverse events in contrast to existing treatments [8, 46]. The current treatment regimen for AC includes non‐pharmacological interventions as the most initial treatment, such as prevention from allergen exposure, use of protective sunglasses, cold compress, isotonic saline drops, and preservative‐free artificial tears. However, these yield only short‐term and incomplete relief in many subjects [2, 3, 4, 5]. These non‐pharmacological interventions provide either only short‐term symptom alleviation, unwanted adverse effects, or a higher number of dosing for a long time eventually resulting in poor patient compliance. Among the first‐line pharmacological treatments available, topical antihistamines, alleviate the inflammation, but only for a short duration, necessitating repeated dosage. Moreover, it has been reported that systemic adverse effects like drowsiness, confusion, and urinary retention are associated with the intake of oral antihistamines. Mast cell stabilizers prove to remain one of the most effective treatments in terms of prophylaxis but cannot be used retroactively for acute symptomatic relief. Thus, all of the above‐mentioned limitations associated with the antihistamines and mast cell stabilizers adversely affect patient compliance. Dual‐acting agents often prescribed as first‐line therapy, provide less frequent dosing and better compliance with optimum results and an improved safety profile, but its ocular side effects can affect its usage [2, 3, 4, 10]. Nonprescription‐based topical decongestants cause vasoconstriction reducing hyperemia and entailing relief, but are deemed unsafe for long‐term use owing to their side effects like rebound hyperemia and tachyphylaxis. Topical NSAIDs are also used for treating the symptoms of AC, however, extended use causes serious adverse effects such as corneal keratitis, ulceration, or perforation rendering it for short‐term relief only. Similarly, due to an increased risk of infections due to immunosuppression, development of cataracts, and raised intraocular pressure, steroids are used for short‐term treatment only, despite providing better relief by lowering mast cell proliferation, inflammatory cytokine production, and cell‐mediated immune responses [2, 3, 4, 10]. Although oral leukotriene inhibitors reduce the symptoms of SAC and PAC; adverse effects, including headaches, neuropsychiatric disorders, cough, and dyspepsia have been reported in individuals with AKC, thereby rendering its limited use [20]. Allergen‐specific immunotherapy is the only long‐term therapy that works by downregulating the Th2 response and overexpressing regulatory T cells, diminishing reactions to allergen exposure. However, the risk of anaphylaxis and long‐term treatment regimen especially in SLIT, raises concerns and the risk of poor compliance [2, 3, 4, 10]. Similarly, the topical calcineurin inhibitors, namely cyclosporine A and tacrolimus, have proved to be effective in the treatment of GPC, VKC, and AKC but their use is associated with stinging sensation in the eyes with increased risk of molluscum contagiosum virus, papillomavirus, or herpesvirus infection. The efficacy of biological agents such as omalizumab and dupilumab for AC remains unclear due to the lack of extensive trials and studies [2]. Surgical interventions can be opted in resistant cases of VLC and AKC, however, the risk of peri‐procedural complications due to the invasive nature and in‐hospital stay, makes these interventions the last resort [2].

Given the adverse effects and limitations of the existing treatment options for AC, Reproxalap stands out as a promising therapeutic alternative and offers a more targeted strategy for reducing ocular inflammation. It has been proven effective therapeutic agent in both early and late phases of allergic response by providing immediate and sustained relief from symptoms of ocular inflammation and thereby making it a valuable alternative for patients with severe or persistent symptoms. Unlike conventional treatment options that carry substantial adverse effects, Reproxalap is well‐tolerated and a safer alternative with no serious adverse events or treatment discontinuations reported in clinical trials. The substantial limitations of the existing treatment have substantially limited the compliance, adherence and patient satisfaction rates. Reproxalap provides a novel treatment option, demonstrating remarkable efficacy and safety profile, which significantly improved patient compliance and adherence. Thus, reproxalap provides new hope in improving the quality of life of AC patients.

## 11 Future Prospects

Reproxalap addresses the long‐term need for novel therapeutic options for AC. The U.S. Food and Drug Administration (FDA) rejected topical ocular Reproxalap to treat signs and symptoms of DED due to limited and ineffective existing treatments, leading to a significant gap in the management of the disease [8, 46]. Aldeyra Therapeutics has announced promising top‐line findings from the phase 3 INVIGORATE‐2 clinical trial of Reproxalap ophthalmic solution in patients with AC. Despite the challenges, Aldeyra defies the odds and submits Reproxalap for reconsideration, demonstrating its efficacy as a therapy for inflammatory ocular surface diseases [54]. The potential of the drug remains evident in the clinical trial that demonstrated the rapid and sustained activity of Reproxalap in diminishing ocular symptoms and redness in patients with AC [8]. However, FDA's decision may lead to comprehensive regulatory approval for Reproxalap in individuals with AC. Therefore, further clinical trials employing robust methodologies with consistent efficacy endpoints and long‐term safety data are crucial to meet the regulatory standards.

The trial's use of clinically relevant thresholds for ocular irritation and redness via the Allergic Conjunctivitis Quality of Life Questionnaire (ACQLQ) marks a notable improvement in our understanding of Reproxalap's therapeutic advantages [44]. Responder analyses also demonstrate its potential to reduce symptoms when compared with vehicles significantly [44]. Furthermore, its unique mechanism of action of modulating RASP indicates a future approach for treating allergic diseases.

Reproxalap exhibits remarkable safety and tolerability. The drug outperforms lifitegrast, used for the treatment of keratoconjunctivitis sicca and DED in terms of the most often reported side effects, enhancing patient adherence and discontinuation rates [55]. The drug's rapid onset of action and brief pain highlight its potential to provide much‐needed relief to patients when given at high doses despite transient increase in symptoms after delivery [44]. This is especially important in conditions like AC, where the burden of symptoms can greatly influence patients' quality of life.

Investigating alternative allergen challenge models to simulate real‐world conditions and validate Reproxalap's efficacy is imperative. Aldeyra Therapeutics is currently undertaking a phase 3 dry eye chamber clinical study to resubmit the New Drug Application (NDA) for Reproxalap for the treatment of dry eye disease [56]. This study, which recruited its first patient in May 2024, is designed to produce useful findings that would allow Aldeyra to resubmit the NDA to the FDA in the second half of 2024. If the study findings are positive, we may expect the NDA resubmission to be examined by the FDA over 6 months, as per FDA instructions, with probable approval by mid‐2025 [56]. Future research studies should focus on explaining patient‐relevant changes in symptoms and better understanding Reproxalap's action. Additionally, RASP concentrations in tears and conjunctivitis of patients with AC hold the potential to understand the treatment response and optimize the therapeutic approach. The possibility of its approval as a therapeutic alternative could be facilitated by further clinical development and testing.

## 12 Limitations

While Reproxalap represents a significant advancement as a novel alternative for AC, it also has certain limitations. The assessment of the long‐term efficacy and safety of Reproxalap is limited, due to the short duration of clinical trials and small sample sizes. There is a need for further longitudinal studies and real‐world data to validate the long‐term efficacy and safety of Reproxalap in diverse populations. Furthermore, discrepancies in patient groups, treatment methods, and environmental factors may limit the applicability of trial results to real‐world settings [8]. Although studies have shown reductions in ocular irritation, its comparative effectiveness to established treatment modalities needs further investigation [8]. The lack of significant differences between Reproxalap concentrations suggests unclear dose‐response relationships requiring more pharmacodynamic investigation. Additionally, the concentration of RASP in tear and conjunctival tissues has not been thoroughly examined, which may influence therapy efficacy assessments [24]. Vigilance and monitoring are essential to maintain safety across diverse patient groups. Additionally, the ocular and systemic side effects reported in the clinical trials highlight the need for post‐marketing surveillance [44]. There is lack of data on drug‐drug interactions of Reproxalap, which is crucial for patients on multi‐drug regimens. The current trials relied on patient‐reported subjective outcomes that are prone to variability and can affect reliability and reproducibility in different studies. Exploring quantifiable biomarkers and objective endpoints can enhance precision in evaluating therapeutic response and clinical effectiveness. Furthermore, there is limited evidence exploring the potential of tachyplaxis with prolonged used of Reproxalap. The cost and availability of Reproxalap as a novel agent may limit its accessibility in low‐resource settings, warranting the need for cost‐effectiveness analyses.

## 13 Conclusion

In conclusion, AC is a prevalent eye disease that poses a significant burden on the population globally. This narrative review emphasizes that in recent years, significant advancements in the understanding and treatment of ocular allergies have occurred, enabling effective and safe management of the majority of the cases. For decades, there have been no new FDA‐approved medications created to address this issue. Although existing treatment approaches are partially effective, they have limitations such as reduced efficacy and adverse effects. In recent clinical trials, Reproxalap has emerged as a novel option for the management of AC, with a remarkable safety and efficacy profile. Its unique mechanism of action, which targets reactive aldehyde species, provides immediate and long‐lasting relief from ocular discomfort. Despite the need for more studies to refine dose regimes and examine long‐term effects, Reproxalap shows the potential to improve patient outcomes. Its potential to fulfill the unmet requirements of patients with AC emphasizes the need for ongoing research and development in this field.

## Author Contributions

**Sanila Mughal:** conceptualization of the study, writing – original draft, writing – review and editing. **Roshanay Ejaz Awan:** conceptualization of the study, writing – original draft, writing – review and editing, visualization. **Hur Abbas:** writing – original draft, writing – review and editing. **Zahra Sania:** writing – original draft. **Ashna Habib:** writing – original draft. **Syeda Kaneez Sakina:** writing – original draft. **Amina Mahmud:** writing – original draft. **Mohammed Mahmmoud Fadelallah Eljack:** supervision, writing – review and editing. All authors have read and approved the final version of the manuscript. The corresponding author takes complete responsibility for the integrity and accuracy of the data.

## Funding

The authors have nothing to report.

## Disclosure

The lead author Mohammed Mahmmoud Fadelallah Eljack affirms that this manuscript is an honest, accurate, and transparent account of the study being reported; that no important aspects of the study have been omitted; and that any discrepancies from the study as planned (and, if relevant, registered) have been explained.

## Ethics Statement

The authors have nothing to report.

## Consent

The authors have nothing to report.

## Conflicts of Interest

The authors declare no conflicts of interest.

## Data Availability

Data sharing is not applicable to this article, as no new data were created or analyzed in this study.
